# Ultrasound-guided lumbar puncture with a needle-guidance system: A prospective and controlled study to evaluate the learnability and feasibility of a newly developed approach

**DOI:** 10.1371/journal.pone.0195317

**Published:** 2018-04-09

**Authors:** Tilo Backhaus, Moritz von Cranach, Jochen Brich

**Affiliations:** Department of Neurology and Neuroscience, Medical Center – University of Freiburg, Freiburg, Germany; University of Toronto, CANADA

## Abstract

**Objective:**

To evaluate the learnability and feasibility of a new technique comprising a needle-guidance-system (NGS) for ultrasound-assisted lumbar puncture.

**Method:**

Using a randomized crossover study design, 24 medical students were asked to perform an ultrasound-assisted lumbar puncture on a gel phantom using two different techniques that each included a paramedian insertion site. Procedure 1 (P1) used a pre-procedural ultrasound scan to predetermine the ideal insertion point. Procedure 2 (P2) applied a new technique comprising an NGS for performing real-time ultrasound-guided lumbar puncture. Success rates and performance times for both procedures were compared. Participants were also asked to complete a post-study questionnaire, both to quantitatively assess the workload involved and state their personal preferences.

**Results:**

In comparison to the pre-procedural scan (P1), the NGS (P2) was associated with a significant increase in the number of successful punctures per participant (5 (P2) [interquartile range: 3.3–5.0] vs. 3 (P1) [interquartile range: 1.3–4.0], p = 0.005), and led to a significant reduction in performance time (118 seconds vs. 80.6 seconds, p < 0.001). In terms of workload perception, NGS use was associated with significantly better performances and lower frustration levels, as rated by students in the post-study questionnaire. Finally, 23/24 participants stated their preference for P2.

**Conclusion:**

Our newly-developed technique for real-time ultrasound-guided lumbar puncture proved to be learnable and feasible for novices, and only required a small amount of training. The use of an NGS therefore has the potential to serve as a key feature of the ultrasound-assisted lumbar puncture.

## Introduction

Lumbar puncture (LP) is an important technical skill commonly performed at the anatomical midline of the spine between the 3^rd^ lumbar and 1^st^ sacral vertebrae using a landmark-guided technique[1]. However, despite being done routinely, the success rates for LP reportedly range from ~80%[2,3]—when multiple skin punctures are allowed—down to 61.5%[4,5] for first attempts. Furthermore, confounding factors such as obesity or advanced age are steadily increasing[1,2].

One way to increase LP success rates is with the assistance of ultrasound. The pre-procedural ultrasound scan, which is used to determine the site of needle insertion, is the approach supported by the largest body of evidence; however, this technique is primarily recommended for complicated punctures[6,7]. Further refinements such as real-time, in-plane approaches remain experimental, mainly due to difficulties in handling[8]. Recently, a new approach combining pre-procedural scanning with paramedian needle insertion resulted in a significant decrease in the number of passes and attempts[9].

Another promising development for ultrasound-assisted punctures are needle-guidance systems (NGSs), which allow visualization of the intended needle route in the tissue. NGSs can potentially accelerate learning curves and increase success rates in different types of punctures[10–18], but only expert case reports and one feasibility study exist for LP [19–21].

The present study aimed to determine how a newly-developed NGS-approach compares to the pre-procedural ultrasound scan technique in terms of success rates, and whether it is a learnable technique for novices. In addition, we quantified the workload and surveyed the perception associated with each of the two techniques. Concluding we can state that our approach proved to be learnable and feasible for novices, and only required a small amount of training.

## Material and methods

### Ultrasound machine and Needle Guiding System

The eZono^®^ 4000 ultrasound machine (eZono AG, Jena, Germany) containing an electromagnetic Needle Guiding System (NGS) known as ‘eZGuide^®^’ was used. The three-dimensional movements of the pre-magnetized needle are displayed in real-time as a colour-coded trajectory on the ultrasound screen. The calculated trajectory then allows the operator to predict the path of the needle through the tissue, even before the skin is punctured. While performing an out-of-plane puncture, the target zone, which represents the intersection of the needle trajectory and the ultrasound beam, is marked with a red square that turns green when it becomes super-imposed with the needle, which means the target zone has been reached.

Prior to the puncture, any needle containing ferromagnetic material can be magnetized under sterile conditions with special magnets (‘eZMag L’ and the ‘eZMag L cap’).

### Phantom models

The procedure for producing a lumbar spine phantom model composed of synthetic ballistic gelatine was based on the protocol described by Morrow et al.[22], and slightly modified for purposes of the present study. The gel phantom was made by embedding a lumbosacral spine model (spine L2-sacrum; Sawbones Europe AB, Malmö, Sweden) in a 10% solution of clear ballistic gelatine (Clear Ballistics, LLC, Fort Smith, AR). The ligamentum flavum/dura complex was represented by a water-fillable spinal gum tube (replacement tube from LP simulator W44031; AKSE GbR, Wiesbaden, Germany) that was placed into the spinal canal; this served to generate tactile feedback and hence inform the student whether the correct target layer had been reached during the course of the puncture. During the trial period, the transparent gel model was covered with black plastic foil so that participants had to exclusively rely on the accompanying ultrasound image to guide the puncture, without any visual or tactile feedback. The spinal tube was placed at depth of 5 cm, which represents the average distance between the skin and the neuraxial structures in humans [6].

### Compared techniques

The eZono^®^ 4000 ultrasound machine with a linear 3- to 12-MHz probe was used for both techniques tested. The NGS was turned OFF for Procedure 1, and ON for Procedure 2. A standard Quincke spinal needle (BD Spinal Needle 22 GA, REF 405256) was used for both procedures. Prior to each of the two procedures, a transverse midline scan was performed to identify the sacrum as well as the spinous processes L2-5, and these were then marked with a white Edding 750 pen by a small line at the long side of the probe. The resulting 5 foil marks represented the anatomical midline and were used to assist orientation in both procedures.

#### Procedure 1: Ultrasound-assisted lumbar puncture (UALP) with pre-procedural scanning

We used a slightly-modified version of the ‘pre-procedure ultrasound-guided paramedian technique’ previously described by Srinivasan et al.[9]

**Fig 1:**
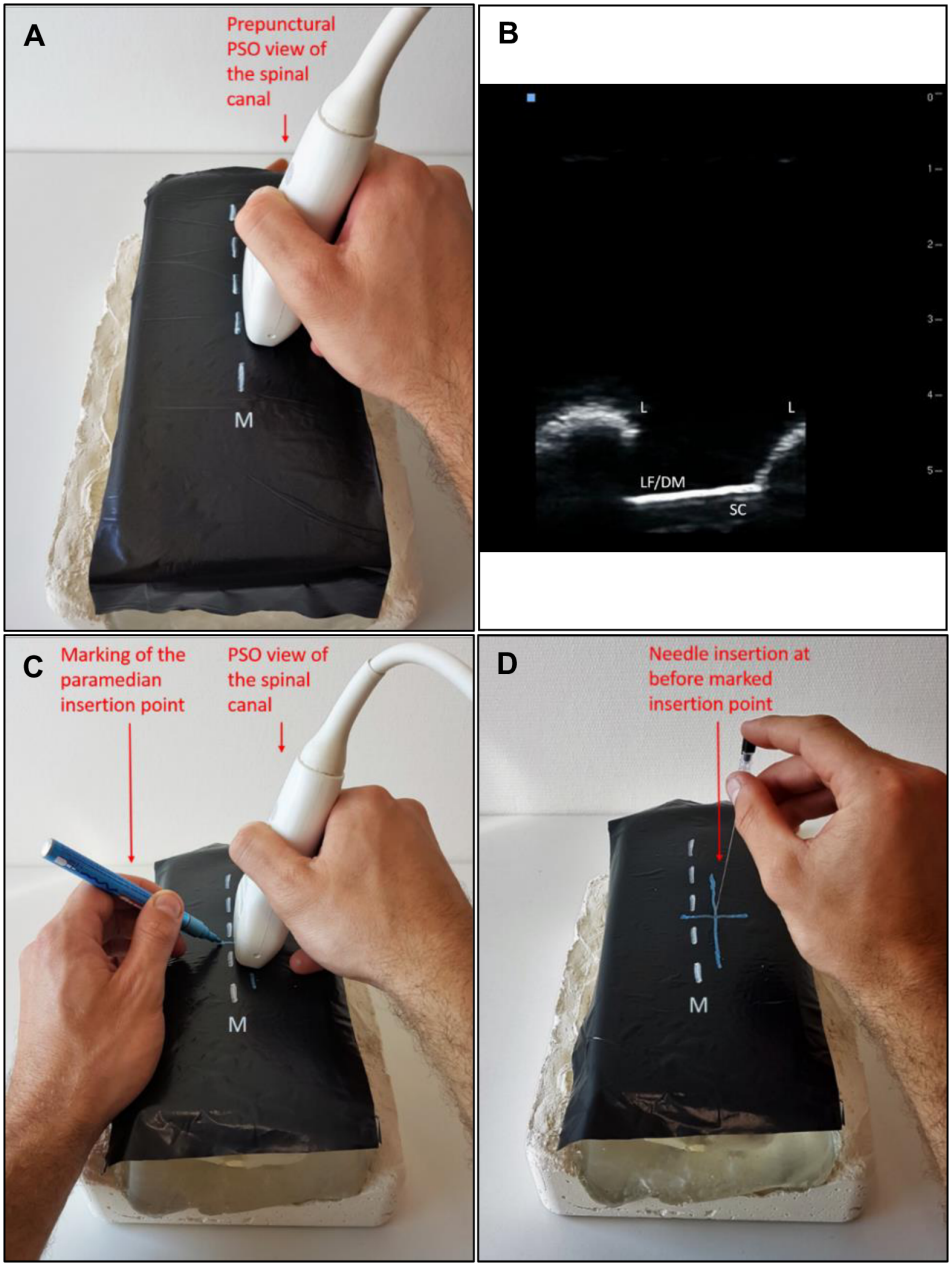
Procedure 1—Puncture with pre-procedural scanning. **A**. PSO view of spinal canal before procedure. **C**. Marking the paramedian insertion point. **D**. Needle insertion at pre-marked insertion point. Steps taken for Procedure 1: The transducer is held in the right (dominant) hand and a paramedian sagittal oblique (PSO) scan is performed by placing the transducer approximately 1 cm lateral to the midline (M) and angling it medially (**A**) until a clear view of the spinal canal (SC) is obtained (**B**). The ligamentum flavum/dura mater complex (LF/DM) is represented by a sharp hyperechoic line in between two adjacent laminae (L). The midpoints of the four borders of the probe can then be marked (**C**). The probe is then set aside and the 4 marks are connected in a cross-wise fashion, thereby defining the needle insertion point. Finally, the needle is inserted with the same angulation used to hold the probe (**D**).

Both the ultrasound scan and the puncture were performed sequentially, each on the same side as the dominant hand (Fig 1). The probe was initially positioned in the sagittal direction, approximately 1 cm lateral to the midline above the sacrum, and angled medially. This plane is often referred to as the paramedian sagittal oblique (PSO)[9] view and provides the best window for ultrasound imaging of the spine[23]. The probe was then moved cranially until the relevant interspace was reached and an optimal view into the spinal canal was obtained. For this view, the tube representing the ligamentum flavum/dura complex should be as visibly clear as possible. With the probe in the correct position, the midpoints of the four borders of the probe were marked with a blue Edding 751 permanent marker. Additionally, the medial angulation of the probe was memorized and later transferred onto the angulation of the needle. The 4 marks were then cross-wisely connected with the intersection, thus defining the paramedian insertion point. Finally, the puncture was performed ‘freehand’ at the marked entry point with respect to both the distance from the drawn midline and the memorized angulation of the probe.

#### Procedure 2: UALP with a NGS

**Fig 2:**
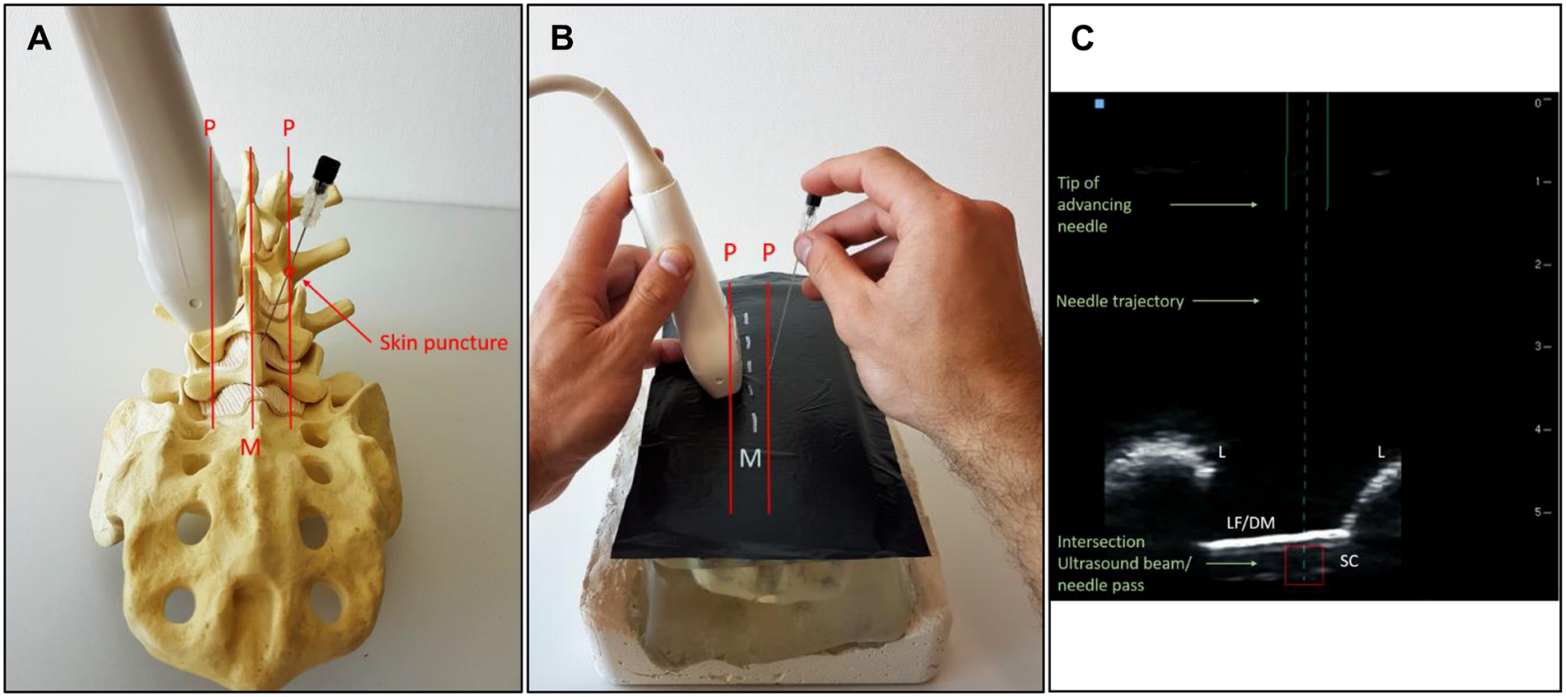
Procedure 2 –Real-time puncture using a needle-guidance-system. Steps taken for Procedure 2: **A**. Schematic overview of the approach to Procedure 2. Both the transducer and the needle are positioned paramedially (P), approximately 1 cm lateral to the midline (M) and with medial angulation. The needle is shown to meet the ultrasound beam near the anatomical midline. **B**. Depiction of Procedure 2 under study-like conditions. The needle is held and introduced with the dominant hand while controlling for its advancement in real-time on the ultrasound screen **(C)**. The solid line in **C** represents the actual advancement of the needle in line with the calculated trajectory, which is represented by the dotted line. The red square embodies the target, namely, the intersection between the ultrasound beam with the needle trajectory. Thus, the target should directly lie beneath the ligamentum flavum/dura mater complex (LF/DM) in the spinal canal (SC) between the two adjacent laminae (L). As soon as the needle (solid line) meets the ultrasound beam, the red square turns green, indicating that the tip of needle is theoretically lying inside the spinal canal.

Both the ultrasound scan and puncture were performed paramedially, approximately 1 cm lateral to the midline on opposite sides (Fig 2). After the midline was marked as described above, right-handers held the probe in their left hand and, beginning above the sacrum, performed a PSO scan on the left side of the midline until they reached the relevant interspinous space. As soon as a clear view of the spinal canal was obtained (see Procedure 1), the magnetized needle was slowly advanced into the magnetic field of the probe, with the dominant hand on the opposite side of the transducer relative to the drawn midline. Now the trajectory of the needle could be seen on the ultrasound screen and positioned centrally in between the adjacent laminae by moving the needle cranially and caudally in the sagittal plane. The needle was then placed gently onto the surface of the phantom, and angled medially until the square box (representing the intersection of the needle trajectory and the ultrasound beam) lay directly beneath the posterior wall of the tube (the ligamentum flavum/dura complex) inside the spinal canal. Afterwards, the needle could slowly be inserted into the phantom while controlling the correct advancement on the ultrasound screen in real-time.

### Study protocol

This randomized, volunteer, crossover study was conducted in the Department of Neurology and Neuroscience at the Medical Center of the University of Freiburg, and approved by the local ethics committee (Ethics Commission of the Medical Center–University of Freiburg; approval number: 427/16). Twenty-four volunteering medical students participating in the regular neurology course were recruited. After written informed consent was obtained, participants were enrolled in six modules in groups of four participants. To assess the novice status of each participant, students were asked to complete a pre-test questionnaire.

A sealed envelope technique was used to pseudo-randomize participants to begin either with Procedure 1 or 2. Each module was split into two consecutive parts, an exercise and a trial section. The one-hour practice period consisted of three parts: Firstly, the participants followed a standardized 10-minute presentation including videos, introducing the objectives of the study and providing the relevant background information for the UALP. This was followed by a 10-minute live demonstration of the two procedures according to standard protocol, and a 40-minute supervised hands-on practice period. During this practice period the participants were divided into pairs, each of whom had 20 minutes to familiarize themselves with both techniques.

In the trial period, the participants were asked to perform six punctures with each procedure, having a small wash-out break after each block of three punctures, and changing to the other modality after six punctures. The punctures were always performed from caudal to cranial in the L4/L5, L3/L4 and L2/3 interspaces, respectively. Successful punctures were first-pass hits only, defined as the backflow of water through the needle after the stylet was removed, without the option of revising the needle position. The number of successful punctures and the time taken between placing the probe onto the surface of the phantom and successfully completing the puncture was noted by the investigators.

### Evaluation

After completion of the 12 punctures, participants were asked to answer a post-study questionnaire containing a modified NASA-TLX, in order to quantify the workload required for each procedure[15,24]. A scale from 0 (low) to 20 (high) was used to rate the perceived amount of work for a specific task across six categories (mental, physical and temporal demand plus the perceived performance, effort, and level of frustration). Additionally, participants were asked to assess their own ability on a scale of 1 (low) to 10 (high), and to state which modality (P1 or P2) they would prefer if they had the choice.

### Statistical analysis

IBM SPSS statistics 23 software (IBM Corp., IBM SPSS Statistics for Windows, Version 23.0. Armonk, NY) was used for statistical analyses. The Wilcoxon signed-rank test for non-parametric paired observations was used to compare the number of successful punctures, performance times and answers to the post-study questionnaire. Differences in success rates based on starting modality or gender were compared using the Mann-Whitney U test. The Spearman rank-order correlation coefficient was used to measure the association between the answers given in the pre-test questionnaire and success rates. The significance level was set at 0.05.

## Results

Demographic data from the 24 participants are presented in Table 1. Most participants had no experience with LP (18/24; 75%) or UALP (22/24; 92%), and less than 10 hours of ultrasound training (19/24; 79%).

**Table 1:** Baseline characteristics of participants.

Variable		N^a^
Gender	Male	11 (46)
	Female	13 (54)
Year of medical degree	3^rd^ year	2 (8)
	4^th^ year	17 (71)
	> 4^th^ year	5 (21)
Experience with lumbar punctures	No experience	18 (75)
	< 10 lumbar punctures on patients or phantoms	5 (21)
	≥ 10 lumbar punctures on patients or phantoms	1 (4)
Experience with ultrasound	< 10 hours training on patients or phantoms	19 (79)
	> 10 hours training on patients or phantoms	5 (21)
	Experienced handling of ultrasound	0 (0)
Experience with UALPs	No experience	22 (92)
	Observation of UALPs	2 (8)
	Execution of an UALP on a patient or phantom	0 (0)
Age		24,40 [21–33] ^b^

Abbreviations: UALP = Ultrasound assisted lumbar puncture

^a^. N = 24 (100%); Data presented as absolute numbers of participants (%)

^b^. Age presented as average age of all participants [range]

The use of the NGS significantly increased the number of successful first pass punctures overall (72% with P2 vs 47% with P1), and per participant (median 5 of 6 with P2 vs 3 of 6 with P1, p = 0.005) (Table 2).

**Table 2:** Successful punctures and time required.

	Preprocedural	Needle guidance	P value ^c^
Successful punctures			
Overall success proportion ^a^	68/144 (47)	103/144 (72)	
Successful punctures per participant ^b^	3 [1.3–4.0]	5 [3.3–5.0]	0.005
Median time to success ^b^	118 [101.7–136.7]	80.6 [73.8–108.0]	< 0.001

Success is presented as number of first pass hits, time is presented in seconds

^**a**^. Values are presented as absolute numbers of first pass hits (%)

^**b**^. Values represent the median [interquartile range]

^**c**^. Two-tailed

NGS application was also associated with a significant reduction in performance time (118 seconds vs. 80.6 seconds, p < 0.001) (Table 2).

Neither the variables from the pre-test questionnaire, nor randomisation to a particular initial modality correlated with the number of successful punctures.

All participants completed the post-study questionnaire. Comparison of the two modalities showed a significant difference in the following three fields, all in favour of the NGS: Participants reported a lower level of frustration (7 vs. 13; p = 0.003), a better self-assessed performance (15 vs. 11; p = 0.004), and an overall higher degree of learnability when using the NGS (7 vs. 4; p = 0.001) (Table 3). These findings also concur with the finding that 23/24 (96%) participants preferred P2 (with NGS) over P1 (pre-procedural scan).

**Table 3:** Results of the post-study questionnaire.

Task question	Pre-procedural scan	Needle guidance	P value ^c^
Mental demand	13.5 (11.0–16.0)	14 (11.0–16.0)	0.819
Physical demand	11.5 (7.5–13.0)	12 (7.0–14.0)	0.393
Temporal demand	10 (6.0–12.0)	8 (5.0–11.0)	0.058
Performance	11 (5.3–14.0)	15 (13.0–17.8)	0.004
Effort	12.5 (11.0–15.0)	12.5 (11.0–15.0)	0.954
Frustration level	13 (7.5–17.0)	7 (4.3–9.0)	0.003
Learner’s assessment of own ability ^a^	4 (3.3–6.0)	7 (6.0–8.0)	0.001
Preferred modality ^b^	1/24 (4)	23/24 (96)	

Data represent the median (interquartile range)

Abbreviations: IQR = interquartile range

^a^. Within a range from 1 (low) to 10 (high)

^b^. Absolute numbers of participants (%)

^c^. Two-tailed

## Discussion

In contrast to other procedures such as central venous line placement into the internal jugular vein[25], ultrasound assistance has not yet made its way into daily neuraxial procedures[26]. This could be due to the extensive training required[27], since envisaging neuraxial structures in 3D in combination with a paramedian or midline puncture is a complex task. Hence, such procedures are usually reserved for experts rather than an inexperienced practitioner who is lacking in confidence.

We considered whether applying an NGS during UALP would help overcome these difficulties. The best approach to NGS-assisted UALP remains unclear due to a lack of systematic evaluations, with only case reports and case series existing. Brinkmann et al.[20] and Niazi et al.[19] agreed that an out-of-plane approach more advantageous to the in-plane approach when using an NGS. We combined this finding with a paramedian puncture technique, and developed a real-time, paramedian, needle-guided out-of-plane puncture (Procedure 2). As the midline marking is not necessarily required for this procedure, only the PSO view needs to be trained, internalized and performed. This not only potentially saves time, it also helps to minimize the levels of background knowledge required for spinal sonoanatomy. Our study evaluated the learnability and feasibility of this new approach.

To systematically examine our new approach, we applied a study framework with three prerequisites:

Firstly, we chose medical students as participants, because they are most likely to be novice operators with minimal experience in ultrasound, LP and UALP. Indeed, the majority of participants were novices in all three modalities, making them ideal candidates for examining both the learnability and feasibility of the two procedures. We chose a certain amount of time for the practice period rather than a specific number of punctures, since, from our perspective, a given time interval better reflects students’ different ways of learning.

Secondly, we used custom-made phantom models, which have the advantage of guaranteeing identical test environments and enabling multiple punctures under the same conditions. Furthermore, phantoms have already been shown to be an appropriate and realistic[28] training tool for learning how to do a LP[29], where the acquired competence can be transferred to a clinical setting[30].

Thirdly, since the conventional landmark-guided approach was not possible in our model due to the lack of a phantom os ilium, we based our procedure on that described by Srinivasan et al.[9] These authors applied a pre-procedural scan using the paramedian sagittal oblique (PSO) view of the spinal canal, which, according to most studies, provides the optimal window for ultrasound imaging[23]. Srinivasan et al. complemented their approach with a paramedian needle insertion point, for which many authors have described theoretical advantages over the conventional midline approach[31,32]. Using this approach in a prospective randomized controlled study, Srinivasan and colleagues were able to demonstrate a significant decrease in the number of both needle passes and attempts in comparison to the conventional landmark-guided midline approach. This combination of paramedian puncture and ultrasound is therefore comparable to our newly-developed P2.

In the present study, this procedure (P1) proved to be fairly learnable by novices and resulted in moderate success rates of 42%, but participants rated it rather highly in terms of frustration levels (median 13/20, IQR 7.5–17) and relatively low for learner assessment of own ability (median 4/10, IQR 3.3–6).

Our newly-developed NGS-assisted approach resulted in a significant increase in successful punctures, as well as shorter performance times compared to the procedure described by Srinivasan et al.[9] Furthermore, the significantly better overall rating for P2, as indicated in the post-study questionnaire, shows the feasibility and good learnability of the procedure, despite it appearing theoretically complex. Participants quickly recognized the osseous structures in the ultrasound image, and then intuitively used the NGS eZGuide^®^ system: Analogous to a simple video game, it urged participants to match the requested target area (i.e., spinal canal) with a virtual red square, and then use the ultrasound screen to insert the needle at a slow pace while controlling for correct advancement in real-time until the square turned green. This visual feedback might be the crucial factor in simplifying the learning process, namely by providing more confidence to the operator and thereby reducing frustration levels; this, in turn, leads to a higher success rate.

Our NGS-assisted approach was learned within just one hour of training, enabling novices to overcome known obstacles of the “blind” paramedian approach, such as an existing understanding of 3D structure and a strong comprehension of sonoanatomy.

Since NGSs are a relatively new development, they are still hampered by the complexity and costs associated with the ultrasound machines and accessories[21]. The electromagnetic NGS used in this study offers two main advantages over other commercially-available solutions: 1. No additional instruments are required because the technology has been incorporated into the transducer, keeping the system simple and familiar, and 2. Most standard LP needles can be used, since they contain ferromagnetic material that is magnetized under sterile conditions prior to the puncture, thus making the procedure cost-neutral.

There were also several limitations to our study. The procedures were conducted on custom-made phantoms composed of synthetic ballistic gelatine, which has a lower echogenity than real tissue. This, however, had no influence on the main outcome of the study, as the same models were used for both procedures. The haptic characteristics of the models and the missing flexion of their spine did not allow a realistic comparison of P2 to the conventional, landmark-guided approach. Since phantom-based studies are generally limited in terms of clinical reality, the clinical relevance of results such as success rates or the duration of a successful puncture is restricted. In addition, results may alter in a clinical setting due to a different handling of the needle and the probe, when the technique is conducted on real patients in the prone, sitting or lateral decubitus position. It has to be noted that some of the participants had a little experience with US, LP and UALP (Table 1). However, we do not think that participants’ previous experience did confound our results substantially, as we applied a cross-over design, ensuring that prior experience, if at all, is potentially useful for both procedures carried out by the same participant. Although the 72% success rate associated with the NGS does not appear advantageous to those rates reported for conventional landmark-guided approaches, it should be noted that this study included absolute beginners who only had about one hour to practice neuraxial ultrasound, using the NGS, and performing the LP itself. More importantly, only first-pass hits were counted as successful. Although first-pass success rates are rarely reported in the literature, those that do exist are just above 60% [4,5]. Accordingly, first-pass hits should set the benchmark for future work, as redirections and osseous contacts tend to cause multiple problems[4,5,33–38] and may be the reason for fear and refusal of LP among patients.

Taken together, we provide the first evidence that an ultrasound-assisted, needle-guided, paramedian, out-of-plane approach significantly enhances LP performance. It reduces the required workload and is easily learned by novices. Although the conventional landmark-guided LP is a relatively safe, easy and cost-effective procedure, UALP with NGS—by making the first-pass hit predictable—could overcome pain, patient discomfort and other complications such as postdural puncture headache, and might therefore become a clinically-plausible alternative to the conventional landmark-guided LP in the future.

Moreover, the use of UALP with NGS could help overcome inexperience and lack of confidence in situations where LP is not performed routinely. It could also serve as an important teaching tool by helping novices to gain a deeper knowledge of spinal anatomy, as well as to gather experience, handling-skills and self-confidence[12,14,39]. Finally, UALP with NGS could serve to either complement—or even replace—fluoroscopically-guided lumbar puncture in difficult LP cases, including those in previously-operated or elderly patients with inflexible spines or difficult landmarks, or in ventilated ICU patients in the prone position. Studies on patients in a clinical setting, comparing P2 to the conventional, landmark-guided technique, are therefore warranted to confirm the promising results of this study, as well as to assess the use of the NGS for the clinical practice.

## Supporting information

S1 File Raw data. (XLSX)

S2 File Modified NASA-TLX. (PDF)

S3 File Modified NASA-TLX–English version. (PDF)
