# Supplementary material for: Ultrasound-guided lumbar puncture with a needle-guidance system: A prospective and controlled study to evaluate the learnability and feasibility of a newly developed approach
Source: PLoS One. 2018 Apr 9;13(4):e0195317. doi: 10.1371/journal.pone.0195317 (PMC5891015; doi:10.1371/journal.pone.0195317)
Supplement: S2 File — (PDF) [file pone.0195317.s002.pdf]

Bitte beantworten Sie die folgenden Fragen und markieren Sie Ihre Antworten auf der Visuellen Analogskale (1-20) mit einem - X -

Probanden-Buchstabe ______

<table>
<tr><th></th><th>Ultraschall mit Nadelführung</th><th>Ultraschall ohne Nadelführung</th></tr>
<tr><td>1. Geistige Anforderung:
Wie hoch waren die geistigen Anforderungen der Aufgabe. War die Aufgabe leicht oder anspruchsvoll, einfach oder komplex, erfordert sie hohe Genauigkeit oder ist sie fehlertolerant?</td><td>|_|_|_|_|_|_|_|_|_|_|_|_|_|_|_|_|_|_|_|_|
Sehr niedrig Sehr hoch</td><td>|_|_|_|_|_|_|_|_|_|_|_|_|_|_|_|_|_|_|_|_|
Sehr niedrig Sehr hoch</td></tr>
<tr><td>2. Körperliche Anforderung:
Wie viel körperliche Aktivität war erforderlich (z.B. drücken, steuern, halten…). War die Aufgabe leicht oder schwer, einfach oder anstrengend, erholsam oder mühselig?</td><td>|_|_|_|_|_|_|_|_|_|_|_|_|_|_|_|_|_|_|_|_|
Sehr niedrig Sehr hoch</td><td>|_|_|_|_|_|_|_|_|_|_|_|_|_|_|_|_|_|_|_|_|
Sehr niedrig Sehr hoch</td></tr>
<tr><td>3. Zeitliche Anforderung
Wie viel Zeitdruck empfanden Sie hinsichtlich der Häufigkeit oder dem Takt mit dem die Aufgaben oder Aufgabenelemente auftraten? War die Aufgabe langsam oder geruhsam oder schnell oder hektisch?</td><td>|_|_|_|_|_|_|_|_|_|_|_|_|_|_|_|_|_|_|_|_|
Sehr niedrig Sehr hoch</td><td>|_|_|_|_|_|_|_|_|_|_|_|_|_|_|_|_|_|_|_|_|
Sehr niedrig Sehr hoch</td></tr>
<tr><td>4. Aufgabenerfüllung
Wie erfolgreich haben Sie Ihrer Meinung nach die vom Versuchsleiter (oder Ihnen selbst) gesetzten Ziele erreicht? Wie zufrieden waren Sie mit Ihrer Leistung bei der Verfolgung dieser Ziele?</td><td>|_|_|_|_|_|_|_|_|_|_|_|_|_|_|_|_|_|_|_|_|
Sehr niedrig Sehr hoch</td><td>|_|_|_|_|_|_|_|_|_|_|_|_|_|_|_|_|_|_|_|_|
Sehr niedrig Sehr hoch</td></tr>
<tr><td>5. Anstrengung
Wie hart mussten Sie arbeiten, um Ihren Grad an Aufgabenerfüllung zu erreichen?</td><td>|_|_|_|_|_|_|_|_|_|_|_|_|_|_|_|_|_|_|_|_|
Sehr niedrig Sehr hoch</td><td>|_|_|_|_|_|_|_|_|_|_|_|_|_|_|_|_|_|_|_|_|
Sehr niedrig Sehr hoch</td></tr>
<tr><td>6. Frustration
Wie unsicher, entmutigt, irritiert, gestresst und verärgert (versus sicher, bestätigt, zufrieden, entspannt und zufrieden mit sich selbst) fühlten Sie sich während der Aufgabe?</td><td>|_|_|_|_|_|_|_|_|_|_|_|_|_|_|_|_|_|_|_|_|
Sehr niedrig Sehr hoch</td><td>|_|_|_|_|_|_|_|_|_|_|_|_|_|_|_|_|_|_|_|_|
Sehr niedrig Sehr hoch</td></tr>
<tr><td>7. Wenn Sie die Wahl hätten, welche Variante würden Sie für die Ultraschall-gesteuerte Lumbalpunktion bevorzugen</td><td>Ultraschall mit Nadelführung ☐</td><td>Ultraschall ohne Nadelführung ☐</td></tr>
<tr><td>8. Schätzen Sie Ihre Fähigkeiten bezüglich der jeweiligen Modalität ein (0-10)</td><td>Ultraschall mit Nadelführung
1 2 3 4 5 6 7 8 9 10</td><td>Ultraschall ohne Nadelführung
1 2 3 4 5 6 7 8 9 10</td></tr>
</table>

Weitere Kommentare (ggf. auf der Rückseite): ____

Erklärungen angelehnt an: https://www.keithv.com/software/nasatlx/nasatlx_german.html
