# Supplementary material for: Ultrasound-guided lumbar puncture with a needle-guidance system: A prospective and controlled study to evaluate the learnability and feasibility of a newly developed approach
Source: PLoS One. 2018 Apr 9;13(4):e0195317. doi: 10.1371/journal.pone.0195317 (PMC5891015; doi:10.1371/journal.pone.0195317)
Supplement: S3 File — (PDF) [file pone.0195317.s003.pdf]

Please answer the following questions and mark your answers on the visual analogue scale (1-20) with an - X -

Test person letter ______

<table>
<tr><th></th><th>Ultrasound with needle guidance</th><th>Ultrasound without needle guidance</th></tr>
<tr><td>1. Mental Demand
How much mental activity was required? Was the task easy or demanding, simple or complex, exacting or forgiving?</td><td>|_|_|_|_|_|_|_|_|_|_|_|_|_|_|_|_|_|_|_|_|
Very low Very high</td><td>|_|_|_|_|_|_|_|_|_|_|_|_|_|_|_|_|_|_|_|_|
Very low Very high</td></tr>
<tr><td>2. Physical Demand
How much physical activity was required (e.g. pulling, controlling, holding…)? Was the task easy or demanding, slow or brisk, slack or strenuous, restful or laborious?</td><td>|_|_|_|_|_|_|_|_|_|_|_|_|_|_|_|_|_|_|_|_|
Very low Very high</td><td>|_|_|_|_|_|_|_|_|_|_|_|_|_|_|_|_|_|_|_|_|
Very low Very high</td></tr>
<tr><td>3. Temporal Demand
How much time pressure did you feel due to the rate of pace at which the tasks or task elements occurred? Was the pace slow and leisurely or rapid and frantic?</td><td>|_|_|_|_|_|_|_|_|_|_|_|_|_|_|_|_|_|_|_|_|
Very low Very high</td><td>|_|_|_|_|_|_|_|_|_|_|_|_|_|_|_|_|_|_|_|_|
Very low Very high</td></tr>
<tr><td>4. Performance
How successful do you think you were in accomplishing the goals of the task set by the experimenter (or yourself)? How satisfied were you with your performance in accomplishing these goals?</td><td>|_|_|_|_|_|_|_|_|_|_|_|_|_|_|_|_|_|_|_|_|
Poor Good</td><td>|_|_|_|_|_|_|_|_|_|_|_|_|_|_|_|_|_|_|_|_|
Poor Good</td></tr>
<tr><td>5. Effort
How hard did you have to work to accomplish your level of performance?</td><td>|_|_|_|_|_|_|_|_|_|_|_|_|_|_|_|_|_|_|_|_|
Very low Very high</td><td>|_|_|_|_|_|_|_|_|_|_|_|_|_|_|_|_|_|_|_|_|
Very low Very high</td></tr>
<tr><td>6. Frustration
How insecure, discouraged, irritated, stressed and annoyed versus secure, gratified, content, relaxed and complacent did you feel during the task?</td><td>|_|_|_|_|_|_|_|_|_|_|_|_|_|_|_|_|_|_|_|_|
Very low Very high</td><td>|_|_|_|_|_|_|_|_|_|_|_|_|_|_|_|_|_|_|_|_|
Very low Very high</td></tr>
<tr><td>7. If you had a choice, which alternative would you choose for the ultrasound-assisted lumbar puncture?</td><td>Ultrasound with needle guidance □</td><td>Ultrasound without needle guidance □</td></tr>
<tr><td>8. Rate your ability for each alternative (0-10)</td><td>Ultrasound with needle guidance
1 2 3 4 5 6 7 8 9 10</td><td>Ultrasound without needle guidance
1 2 3 4 5 6 7 8 9 10</td></tr>
</table>

Further comments (if necessary on the back): ____

Explanations from: http://keithv.com/software/nasatlx/nasatlx.html
